# Herbal remedies used for the treatment of infertility in males and females by traditional healers in the rural areas of the West Bank/Palestine

**DOI:** 10.1186/s12906-019-2617-2

**Published:** 2019-07-31

**Authors:** Nidal Jaradat, Abdel Naser Zaid

**Affiliations:** Department of Pharmacy Faculty of Medicine and Health Sciences, An-Najah National University, P.O. Box 7, Nablus, Palestine

**Keywords:** Herbal remedies, Traditional healers, Infertility, West Bank/Palestine

## Abstract

**Background:**

Infertility is considered one of the global public health problems and during human history, it is also considered one of the unsolved problems of the continuous human race. This study aimed to collect and document the ethnopharmacological data on herbal remedies, which traditionally used by Palestinian healers in the rural areas of the West Bank area for the treatment of infertility in males and females.

**Methods:**

Using a semi-structured questionnaire, an ethnopharmacological survey of medicinal plants used for the treatment of infertility in the West Bank area of Palestine was investigated. This survey involved 51 traditional healers which were interviewed in rural areas from 9 Palestinian regions.

**Results:**

Information about 31 plants used in the treatment of infertility in females and 24 plants used in the treatment of infertility in males were collected. This information including names of plants, parts used, mode and methods of preparation which were obtained from 51 traditional healers interviewed in rural areas of 9 regions of the West Bank/Palestine. This investigation is the first scientific work in the Middle East area which collected information about herbal remedies used by local Palestinian traditional healers for the treatments of infertility in males and females. The highest Frequency of Citation (FC) of herbal remedies used in case of infertility in females, were 98.04% for pollen grains from *Ceratonia siliqua*, 88.24% for *Anastatica hierochuntica* fruits and 84.31% for *Parietaria judaica* leaves, while the highest Frequency of Citation (FC) of herbal remedies used in case of infertility in males were 96.08% for *Ferula hermonis* roots, 88.24% for *Phlomis brachyodon* leaves and 86.27% for *Phoenix dactylifera* pollen grains.

**Conclusion:**

Herbal healers in the West Bank area of Palestine have a wide range of herbal remedies used in case of infertility in males and in females. Unfortunately, most of them lack scientific evidence of pharmacological or toxicological nature. Therefore, the information obtained in this study can serve as a scientific base for further investigations to determine their efficacy and safety which might contribute to better integration of Palestinian traditional medicine into the global health system in the future.

## Background

Ethnopharmacological surveys have been found to be one of the most reliable tools for the discovery of the natural and semi-synthetic drug. In fact, herbals and other natural products, including their chemical derivatives, represented about 50% of all currently utilized medications worldwide [1]. The usage of plants Kingdom by human beings, as a source of medicines, started from the immemorial time for treatment, protection, and prevention of various illnesses. Till recent time, herbals are considered one of the most important branches of traditional medicine. In fact, this kind of medicine plays until now an important role in health care systems, especially in rural areas in developed and developing countries [2–6]. In fact, traditional medicine is considered a very important branch of pharmacy and medicine and besides that, the used plants in this medicine are considered major sources for the investigation of pharmacologically active drugs in the pharmaceutical industry. In addition, the global public interests are in continuous growing toward the use of this type of medicine. In fact, about 80% of people in rural areas of developing countries utilized traditional medicine, since it is available, cheap and has a variety of health benefits [7–9].

In Palestine and other countries, traditional healers are well-known by different names such as traditional medical practitioners, traditional doctors, people’s doctors, healers, practitioners of Arabian traditional medicine, Arabian therapists, therapists in prophet medicine, and Islamic practitioner healers [10].

According to the World Health Organization and the International Committee for Monitoring Assisted Reproductive Technology, infertility is a disorder of the reproductive system which is defined by the failure to achieve a clinical pregnancy after one year or more of regular unprotected sexual intercourse [11].

Recently, a huge number of factors caused an increase in infertility levels among males and females, especially in developed countries. These factors include the increased use of contraceptives, rising maternal age, smoking, alcohol, genetic factors, pesticides, narcotics, rates of abortion, and critical economic situations. Besides that, an increase in male impotency could be due to the psychogenic factors, vascular disturbances, neurogenic disorders, endocrine system disturbances and drug treatment [12, 13]. This health care problem can lead to serious psychological disorders, severe stressful and depressing life for parents. In fact, this global problem ranked in the fourth position after the death of the mother, the death of father and unfaithfulness of partner [14]. Accordingly, the treatment of infertility has become a large pharmaceutical and medical industries issues, arranging from manufacturing and prescribing fertility hormones and other drugs to in vitro fertilization operations [15]. In 2010, the World Health Organization (WHO) estimated that about 48.5 million couples worldwide were infertile and 1.9% of women aged 20–44 who wanted a child were unable to have their first live birth. In addition, 10.5% of women who had previously given birth were unable to have another baby after five years of trying. This may raise the question about the used measures that caused this high percentage [14, 16, 17].

In the West Bank area, the infertility rate among men and women is relatively high with a rate of about 15% in 2016 according to World Health Organization, the Sixty-ninth World Health Assembly report [18].

The treatment of infertility in males and females are varying in their associated risks, intensity, and invasiveness which depend on the duration, cause, age, and personal preferences. Meanwhile, the physical, financial, and time commitment is the required factors for infertility treatment. The infertility treatments can range from medication therapy to induce ovulation to invasive manipulation of eggs and sperm outside of the body [19]. As well as all the infertility treatments are very expensive, many of poor patients or patients who believe in alternative medicine or others people which their previous infertility treatments had failed, all of those are seeking for the alternative herbal medicine to solve this problem [20].

Throughout this ethnopharmacological survey, the current study aimed to collect information about herbal remedies used by local rural traditional healers in 9 regions of the occupied West Bank Area of Palestine which used in the treatment of infertility in males and females. The collected data including the plant’s names, used parts, methods of preparations and route of administrations.

## Materials and methods

### Study areas

Palestine has been the battleground of the great powers and civilizations in the region throughout its history, which occurred due to its specific location at the crossroads of Africa, Asia, and Europe, Conquerors of the region included Egypt, Assyria, Macedonia, Rome, Byzantium, Arabia, and Turkey. Settlement in the area is believed to date back to about 8000 B.C.E., to the village of Jericho in the West Bank. West Bank area is one of the important parts of historical Palestine (Holy Land), which considered holy by Jews, Christians, and Muslims. Part of the significance of the land stems from the religious significance of Jerusalem, the historical region of Jesus’ ministry, the holiest city to Judaism, and the site of the Isra and Mi’raj event in Islam. Accordingly, this region represents a very important source of information for the field of ethnomedicine. Due to specific geographical location and climatic conditions (mostly Mediterranean), West Bank area of Palestine is a suitable place for growing a huge variety of herbals. Nowadays, of about 2700 plant species which have been identified in this country [21].

The West Bank is an area of extensive wild biodiversity, farming, and valuable rangelands.

Its central mountain chain endowed with a mild climate is grooved by deep valleys, rich in natural resources, and stretches into rolling hills that plunge further east into the Jordan Valley and the Dead Sea which considered the lowest point on earth and has a worldwide attraction [22]. Due to its distinctive geographical location, climatic diversity, historical profile, and religious variables, all these factors affected positively and enriched the diversity of ethnomedicine in this small area of the world [23].

### Data collection from traditional healers

**Fig. 1:**
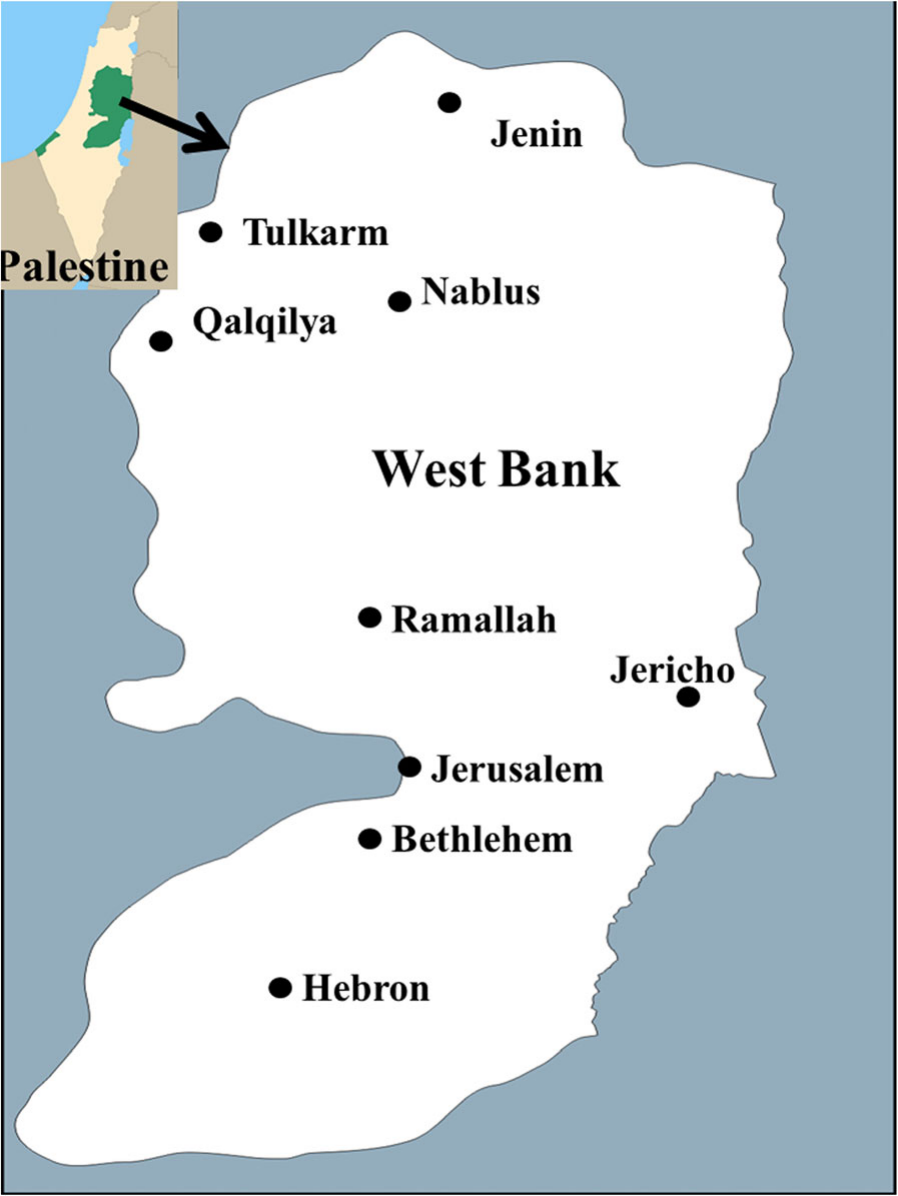
Map of the West Bank/Palestine showing all surveyed areas

Ethnopharmacological data about herbal remedies used in the treatment of infertility in males and females were collected through open-ended semi-structured interviews with 51 traditional healers (informants) during fieldwork. The interviews were conducted in the Arabic language which is the native language of the informants. This survey was conducted between June and August 2017. During the transect walks, plants were collected under the supervision of the traditional healers. Plant voucher specimens were collected and deposited at the Natural products Laboratory, An-Najah National University for identification. Plant names have been checked and updated with the online website (www.theplantlist.org) of the Royal Botanic Gardens, Kew, accessed on 3 July 2017. Areas visited included some rural regions of the West Bank/ Palestine, including rural areas of Nablus, Jenin, Tulkarem, Qalqilya, Ramallah, Jericho, Jerusalem, Bethlehem, and Hebron regions (Fig. 1).

The study protocol and the informed consent forms were approved by the Institutional Review Board (IRB) at An-Najah National University (Archived number 9th June 2017). The study was conducted in accordance with the requirements of the declarations of Helsinki (World Medical Association 2008), Harmonization (ICH1996) Guidelines, the current Good Clinical Practice (GPC) Guidelines (EME 1997) and the International Conference and written informed consent for participation in the study was obtained from all informants.

To protect the interest of traditional healers, they were informed by the researchers in details about the current study and its purposes, also they were not offered any incentives and they were able to withdraw from this study at any time.

The survey was carried out by using interviews among 51 traditional healers (*N* = 51) from different rural areas of Nablus, Jenin, Tulkarem, Qalqilya, Ramallah, Jericho, Jerusalem, Bethlehem and Hebron regions in the West Bank/Palestine. These traditional healers were well-known in the various Palestinian communities and herbalists which utilized herbal products to treat infertility.

The plant’s vernacular names, methods of preparation and administration of these herbal remedies were collected throughout these interviewees (Social demographic factors are presented in Table 1).

**Table 1:** Social-demographic factors related to the informants

Variable	Number of folkloric healers (*N* = 51)	%
Gender, %		
Male	23, (45.1%)	45.1
Female	28, (54.9%)	54.9
Education level, %		
Uneducated	52.9	52.9
Elementary school	25.5	25.5
Secondary school	21.6	21.6
Residency		
Bethlehem region rural areas	9	17.6
Hebron region rural areas	7	13.7
Jenin region rural areas	3	5.9
Jericho region rural areas	16	31.4
Jerusalem region rural areas	5	9.8
Nablus region rural areas	3	5.9
Qalqilya region rural areas	3	5.9
Ramallah region rural areas	3	5.9
Tulkarem rural areas	2	3.9
Age (mean ± SD) years	53.5 (19.6)	
Years of experience as a healer		
Between 1 and 10 years	1	2.0
Between 11 and 20 years	8	15.7
Between 21 and 30 years	7	13.7
Between 31 and 40 years	27	52.9
Between 41 and 50 years	6	11.8
More than 51 years	2	3.9
The sources of traditional healer acquired knowledge		
Ancestors	48	94.1
Books and magazines	2	3.9
Internet	1	2.0

### Data analysis

The Choice Value (CV) method is a valuable assessment tool to measure related plant species for the treatment of infertility in males and females [24].

The CV is calculated as in the following equation:

$$ CVspecies=\frac{Pcs}{Sc}\ \mathrm{x}\ 100 $$

*Pcs*: percent of informants that cited certain plant species for the treatment of infertility in males and females.

*Sc*: is the total number of species mentioned for the treatment of disease by all informants. Choice values are ranked from 0 to 100 with 100 indicating complete preference and fewer alternatives.

The frequency of citation (FC) for all plants species in this study was calculated by using the following formula [25]:

$$ \mathrm{FC}=\left(\mathrm{Number}\ \mathrm{of}\ \mathrm{times}\ \mathrm{a}\ \mathrm{particular}\ \mathrm{species}\ \mathrm{was}\ \mathrm{mentioned}\ \mathrm{by}\ \mathrm{traditional}\ \mathrm{healers}/\mathrm{a}\ \mathrm{total}\ \mathrm{number}\ \mathrm{of}\ \mathrm{occasions}\ \mathrm{that}\ \mathrm{a}\mathrm{ll}\ \mathrm{species}\ \mathrm{were}\ \mathrm{mentioned}\right)\ \mathrm{x}\ 100 $$

## Results

### Social demographic informant’s details

The results of social and demographic factors of informants showed that the percentage of female healers is slightly higher than males and most of them were uneducated. Precisely, the selected 51 traditional healers from various rural areas of West Bank/Palestine comprised 45.1% of the males and 54.9% females. The majority of traditional healers were uneducated 52.9%, while the elementary and secondary schools’ education levels represented 25.5 and 21.6%, respectively (Table 1). The highest percent of informants was from Jericho rural area followed by Bethlehem and Hebron which represented 17.6 and 13.7%. In addition, the majority of them (52.9%) had experienced between 31 and 40 years as well as the most important thing in this survey, 94.1% of these traditional healers acquired their knowledge from their ancestors.

## Results and discussion

The results of the present study showed that a total of 31 plants from 24 families used for the treatment of infertility in females, meanwhile 24 plants from 20 families used for the treatment of infertility in males in West Bank area of Palestine as shown in Tables 2 and 3.

**Table 2:** The used herbals for the treatment of infertility in females, the plant’s parts used, Frequency of Citation (FC), Choice values, modes of administration and methods of preparation

Scientific names	Local names	English names	Family	Voucher number	Part used and mode of preparation	Method of preparation	Number of citations	FC, %	CV
*Ceratonia siliqua* L.	خروب	Locust bean	Leguminosae	Pharm-PCT-584	Pollen grains/About 0.2 g of the pollen grains inhaled 2 h before sexual intercourse.	Powder	50	98.04	3.16
*Anastatica hierochuntica* L.	كف مريم	Rose of Jericho or Dinosaur plant	Brassicaceae	Pharm-PCT-156	Fruits/Soak 50 g of the crushed fruits in 500 ml water. 100 ml of this infusion is to be given once daily for 7 days after the finishing of menopause.	Infusion	45	88.24	2.85
*Parietaria judaica* L.	حشيشة الزجاج	Spreading pellitory or Pellitory of the wall	Urticaceae	Pharm-PCT-1790	Leaves/Boil about 15 g of the leaves in 150 ml water. About 50 ml of this decoction is to be given orally before each meal.	Decoction	43	84.31	2.72
*Saussurea costus* (Falc.) Lipsch.*	القسط الهندي	Costus or kuth	Compositae	Pharm-PCT-2800	Roots/Boil about 50 g of roots in 250 ml water for 15 min. This decoction is to be given three times a day after meals.	Decoction	41	80.39	2.59
*Peganum harmala* L.	الحرمل	Syrian Rue	Nitrariaceae	Pharm-PCT-1801	Fruits/Boil 10 g of the fruits in 500 ml water and used intensively as vaginal douche.	Decoction	39	76.47	2.47
*Phoenix dactylifera* L.	تمر	Date or date palm	Arecaceae	Pharm-PCT-1842	Fruits/Take two of Date fruits and boil them in water (about 50 ml) and the produced solution is to be mixed with equal quantities of Olive oil and Honey. This mixture to be taken early morning once daily.	Decoction	34	66.67	2.15
*Ankyropetalum gypsophiloides* Fenzl	شرش حلاوه	Anchor capillary	Caryophyllaceae	Pharm-PCT-172	Flowers/Steep 1 g of the flowers in 50 ml of fresh Donkey’s milk for 6 h. This infusion is to be given once daily.	Infusion	25	49.02	1.58
*Trigonella foenum-graecum* L.	الحلبة	Fenugreek	Leguminosae	Pharm-PCT-2778	Seeds/Mix 50 ml of the oil of the seeds with equal amounts of royal jelly. One tablespoon of this mixture is to be given once daily before sexual intercourse	Paste	25	49.02	1.58
*Viscum cruciatum* Sieber ex Boiss.	دبق الاحمر	Red-berry mistletoe	Santalaceae	Pharm-PCT-2662	Leaves/About 5 g of dried and ground leaves are to be given orally twice daily with a cup of water.	Powder	25	49.02	1.58
*Cheilocostus speciosus* (J.Koenig) C.D.Specht	القسط	Crepe ginger	Costaceae	Pharm-PCT-2799	Rhizomes/Powdered rhizomes mixed with equal amounts of Royal Jelly. This mixture is to be given before meals three times daily.	Paste	25	49.02	1.58
*Nigella sativa* L.	قزحه	Black cumin	Ranunculaceae	Pharm-PCT-2797	Seeds/Boil 30 g of the seeds with 100 ml water. This decoction is to be orally given before sexual intercourse.	Decoction	24	47.06	1.52
*Artemisia judaica* L.	البعيثران	Judean wormwood	Compositae	Pharm-PCT-238	Flowers/Boil 50 g of the flowers in 500 ml water and used as vaginal douche before sexual intercourse.	Decoction	24	47.06	1.52
*Conium maculatum* L.	الشوكران	Hemlock	Apiaceae	Pharm-PCT-657	Fruits/Steep 0.5 g of the flowers with 500 ml water for 5 h. About 20 ml of this infusion is to be given orally three times a day.	Infusion	22	43.14	1.39
*Ficus carica* L.	التين	Common fig	Moraceae	Pharm-PCT-1028	Leaves/Boil the fresh leaves (about 50 g) with water and the produced decoction is mixed with few drops of the Wolf bile and used as vaginal douche	Decoction	21	41.18	1.33
*Prunus mahaleb* L.	المحلب	St. Lucie cherry	Rosaceae	Pharm-PCT-2798	Seeds/Steep about 50 g of the seeds in 100 ml goat milk for three hours. This infusion is to be given after each meal.	Infusion	21	41.18	1.33
*Ferula orientalis* L.	الكلخ الشرقي	Samaria giant fennel	Apiaceae	Pharm-PCT-1020	Fruits/About 20 g of the powdered seeds soaked one night in olive oil. The produced mixture is to be given three times daily during the menstrual period.	Infusion	20	39.22	1.27
*Juglans regia* L*.*	الجوز	Persian walnut, English walnut,	Juglandaceae	Pharm-PCT-2714	Bark/Boil 200 g from the ground bark in 800 ml water for 30 min. 400 ml of this decoction is to be used as vaginal douche twice daily.	Decoction	20	39.22	1.27
*Sesamum indicum* L.	السمسم	Sesame	Pedaliaceae	Pharm-PCT-2722	Seeds/Handful of grounded seeds is to be eaten once daily.	Powder	19	37.25	1.20
*Quercus coccifera* L.	بلوط قلبريني	Palestine oak	Fagaceae	Pharm-PCT-1978	Bark/Steep 100 g from the bark in 500 ml water for 20 min. 250 ml of this decoction is to be used as vaginal douche twice daily.	Infusion	19	37.25	1.20
*Convallaria majalis* L.	زنبق الوادي	Lily of the Valley	Asparagaceae	Pharm-PCT-2796	Flowers/Soak 10 g of the flowers in 500 ml of the horse milk or donkey milk for 3 h This infusion is to be given orally three times daily after the menstrual period.	Infusion	19	37.25	1.20
*Alchemilla vulgaris* L.	رجل الأسد	Common lady’s mantle	Rosaceae	Pharm-PCT-2801	Leaves/Steep about 30 g of the leaves in 150 ml water for two hours. This infusion is to be given after each meal.	Infusion	18	35.29	1.14
*Salvia fruticosa* Mill.	مريميه	Sage	Lamiaceae	Pharm-PCT-2117	Fruits/About 50 g of the crushed fruits soaked one night in water. The produced infusion used as vaginal douche twice daily.	Infusion	17	33.33	1.08
*Chrozophora tinctoria* (L.) A.Juss.	الغبيراء	Dyer’s croton or turnsole	Euphorbiaceae	Pharm-PCT-611	Leaves/About 10 g of grounded leaves mixed with 10 ml Cow’s bile to produce a thick solution. This mixture diluted with water which used as vaginal douche once daily before bedtime.	Paste	17	33.33	1.08
*Clematis flammula* L.	ظيان شعلي	Fragrant virgin’s bower	Ranunculaceae	Pharm-PCT-631	Flowers/Soak about 10 g of the flowers in 50 ml of water for 12 h. This infusion is to be given five times a day after meals.	Infusion	17	33.33	1.08
*Juniperus drupacea* Labill.	عرعر سوري	Syrian juniper	Cupressaceae	Pharm-PCT-1296	Fruits/Soak 200 g in 800 ml water. About 450 ml of this decoction is to be given orally twice daily before meals.	Infusion	13	25.49	0.82
*Origanum jordanicum* Danin & Kunne	مردقوش شائع	Jordan Thyme	Lamiaceae	Pharm-PCT-1729	Flowers/Boil about 100 g of the flowers in 500 ml water for 25 min. This decoction is to be given five times a day after meals.	Decoction	13	25.49	0.82
*Ricinus communis* L.	حب الخروع	Castor	Euphorbiaceae	Pharm-PCT-2742	Seeds/One Castor seed soaked one night in 200 ml water. The produced infusion used as vaginal douche before intercourse.	Infusion	13	25.49	0.82
*Quercus infectoria* subsp.veneris (A.Kern.) Meikle	بلوط حلبي	Aleppo Oak	Fagaceae	Pharm-PCT-1977	Bark/Boil 150 g from the ground bark in 200 ml water for 20 min. 50 ml of this decoction is to be used as vaginal douche twice daily.	Decoction	13	25.49	0.82
*Rosmarinus officinalis* L.	اكليل الجبل	Rosemary	Lamiaceae	Pharm-PCT-2732	Leaves/Boil about 50 g of the leaves in 500 ml water for 10 min. 100 ml from this decoction is to be used as vaginal douche before each sexual intercourse.	Decoction	11	21.57	0.70
*Syzygium aromaticum* (L.) Merr. & L.M.Perry	كبش قرنفل	Clove	Myrtaceae	Pharm-PCT-2767	Flowers buds/Steep one gram of the buds in100 ml hot water for 12 h. The produced infusion is to be used as vaginal douche before sexual intercourse	Infusion	9	17.65	0.57
*Crocus sativus* L.	زعفران	Saffron	Iridaceae	Pharm-PCT-2733	Flowers/Boil about 2 g of the flowers in one cup of milk for 10 min. Gives 50 ml of this decoction orally every day during the menstrual period.	Decoction	4	7.84	0.25

**Table 3:** The used herbals for the treatment of infertility in males, the plant’s parts used, Frequency of Citations (FC), Choice values, modes of administration and methods of preparation

Scientific names	Local names	English names	Family	Voucher numbers	Part used and mode of preparation	Method of preparation	Citations	FC, %	CV
*Ferula hermonis* Boiss.	شرش الزلوع	Hermon ferula	Apiaceae	Pharm-PCT-1018	Roots/About 50 g of the roasted seeds soaked in 330 ml water for 12 h. The produced infusion is to be given twice a day.	Infusion	49	96.08	4.00
*Phlomis brachyodon* (Boiss.) Zohary ex Rech.f.	القصعين (الأذينة قصيرة الأسنان)	Short-toothed phlomis	Lamiaceae	Pharm-PCT-1832	Leaves/Boil about 50 g of leaves in 750 ml water for 5 min. 150 ml of this decoction is to be given orally before each meal.	Decoction	45	88.24	3.68
*Phoenix dactylifera* L.	تمر	Date or Date Palm	Arecaceae	Pharm-PCT-1842	Pollen grains/About 1 g of the Date trees pollen grains mixed with one 1 g of royal jelly. This paste to be given 2 h before each sexual intercourse.	Paste	44	86.27	3.59
*Luffa cylindrica* (L.) M.Roem.	الليف	Sponge gourd or Egyptian cucumber	Cucurbitaceae	Pharm-PCT-2806	Fruits/About 5 ml of fruits juice to be given twice daily for two weeks	Juice	40	78.43	3.27
*Adonis aestivalis* L.	عين الديك	Summer pheasant’s-eye	Ranunculaceae	Pharm-PCT-23	Leaves/Soak 5 g of the leaves in 400 ml water for 12 h and drink once daily.	Infusion	35	68.63	2.86
*Ferula communis* L.	الكلخ الشائع	Common giant fennel	Apiaceae	Pharm-PCT-1016	Rhizomes/Mix 200 g of the rhizomes with 200 g of dried locust insect and 150 ml of olive oil. About 50 g of the produced paste is to be given orally two times daily.	Paste	31	60.78	2.53
*Myristica fragrans* Houtt.	جوز الطيب	Nutmeg	Myristicaceae	Pharm-PCT-2716	Seeds/Two drops of the oil of the seeds are to be given with one cup of the Camel milk before sexual intercourse.	Oil	26	50.98	2.12
*Pinus halepensis* Mill.	صنوبر	Aleppo pine	Pinaceae	Pharm-PCT-1863	Leaves/Boil about 100 g from the leaves in 100 ml water. 50 ml from this decoction is to be given 6 times daily.	Decoction	24	47.06	1.96
*Eruca sativa* Mill.	جرجير	Arugula	Brassicaceae	Pharm-PCT-2786	Seeds/Boil 50 g of the seeds in 100 ml water for 10 min. About 20 ml of this decoction is to be given orally four times daily.	Decoction	24	47.06	1.96
*Cnicus benedictus* L.	قنطريون مبارك	St. Benedict’s thistle	Compositae	Pharm-PCT-639	Roots/Boil 100 g from fresh roots in 330 ml water for 10 min. 100 ml of this decoction is to be given orally three times daily after meals.	Decoction	23	45.10	1.88
*Mandragora officinalis* Mill.	تفاح المجن	Mandrake or Satan’s apple	Solanaceae	Pharm-PCT-1509	Roots/Mix equal amounts of the roots powder with honey. 30 g of this paste is to be taken orally twice daily.	Paste	23	45.10	1.88
*Lepidium sativum* L	حب الرشاد	Cress	Brassicaceae	Pharm-PCT-2802	Seeds/Boil 100 g of the seeds in 500 ml water for 15 min. About 100 ml of this decoction is to be given orally once a day.	Decoction	22	43.14	1.80
*Rumex cyprius* Murb.	حميض	Knotweed	Polygonaceae	Pharm-PCT-2070	Leaves/Fresh leaves juice is given four times daily (about 30 ml each time).	Juice	22	43.14	1.80
*Zingiber officinale* Roscoe	زنجبيل	Ginger	Zingiberaceae	Pharm-PCT-2724	Rhizomes/Mix 75 g of the roasted rhizomes with 50 g of honey. One tablespoon of this paste is to be given for patient one hour before sexual intercourse.	Paste	21	41.18	1.72
*Raphanus raphanistrum subsp. sativus* (L.) Domin	فجل	Cultivated radish	Brassicaceae	Pharm-PCT-2770	Seeds/Mix equal amounts of the crushed seeds and honey. One tablespoon of this paste is to be given once daily.	Paste	20	39.22	1.63
*Cucurbita maxima* Duchesne	قرع	Atlantic giant pumpkin	Cucurbitaceae	Pharm-PCT-2762	Seeds/Boil 100 g of the crushed seeds in 350 ml water and drink this decoction once daily.	Decoction	20	39.22	1.63
*Portulaca oleracea* L.	فرفحينه	Common purslane	Portulacaceae	Pharm-PCT-1935	Leaves/Soak about 150 g of the crushed leaves in 500 ml water. This infusion is to be given after each meal.	Infusion	19	37.25	1.55
*Allium cepa* L.	بصل	Common onion	Amaryllidaceae	Pharm-PCT-2703	Bulb/Mix 20 ml of onion juice with 5 ml apple vinegar. This mixture is to be given once daily in the early morning time.	Juice	18	35.29	1.47
*Ziziphus spina-christi* (L.) Desf.	سدر	Sidr	Rhamnaceae	Pharm-PCT-2693	Flowers/Boil 1 g of the flowers in 100 ml water and drink this decoction once daily.	Decoction	17	33.33	1.39
*Urtica urens* L.	القريص	Small nettle	Urticaceae	Pharm-PCT-2562	Pollen grains/Boil 10 g of the powdered pollen grains in 50 ml water for 10 min. The produce decoction is to be mixed with royal jelly (equal amounts) and given orally before sexual intercourse.	Decoction	13	25.49	1.06
*Lens culinaris* Medik.	عدس	Lentil	Leguminosae	Pharm-PCT-2805	Seeds/Boil 100 g of the ground seeds in 200 ml water and 50 ml olive oil for 20 min. 100 ml of this decoction is to be given twice daily	Decoction	13	25.49	1.06
*Cyperus esculentus* L.	حب العزيز	Nut grass or earth almond	Cyperaceae	Pharm-PCT-2803	Roots/Soak 100 g of the roots in 800 ml of water for one night. The produced infusion is to be given 3–5 times a day.	Infusion	11	21.57	0.90
*Hordeum spontaneum* K.Koch	شعير بري	Wild barley	Poaceae	Pharm-PCT-1211	Seeds/Boil 100 g of the crushed seeds in 100 ml water and drink twice daily after meals.	Decoction	10	19.61	0.82
*Anacardium occidentale* L.	كاشو	Cashew	Anacardiaceae	Pharm-PCT-2804	Seeds/Steep 50 g of the grounded seeds in 100 ml water for 6 h. About 20 ml from this infusion is to be given three times a day.	Infusion	5	9.80	0.41ii

* This plant should not be used according to Species at Risk of Extinction (https://portals.iucn.org/library/efiles/documents/PP-003-En.pdf)

**Fig. 2:**
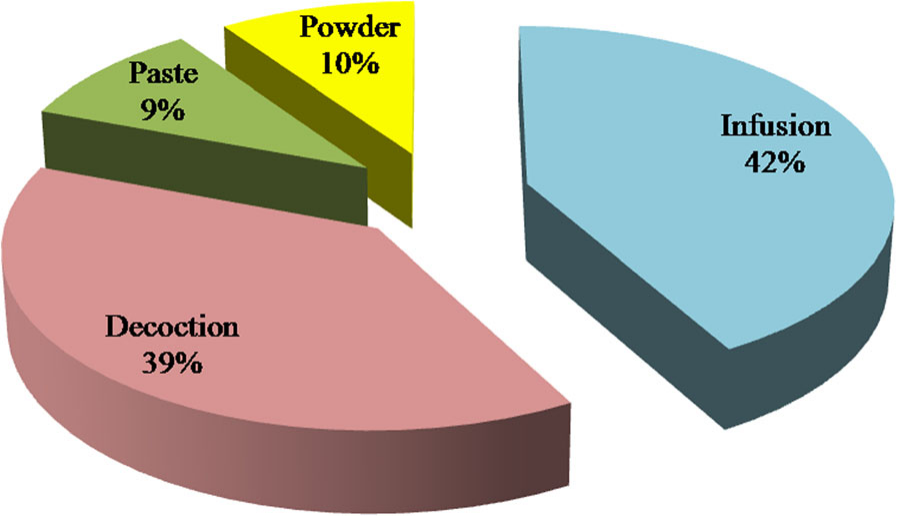
Frequency of herbal remedies preparation methods which used for the treatment of infertility in females

**Fig. 3:**
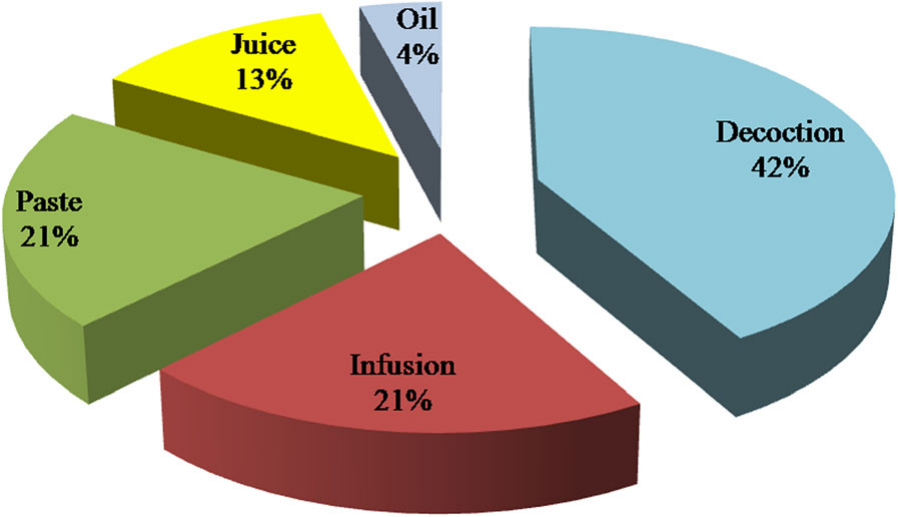
Frequency of herbal remedies preparation methods which used for the treatment of infertility in males

Infusions and decoctions were the most frequently used methods of preparation for treatment of infertility in females as presented in Fig. 2. Meanwhile, decoctions and infusions were the most frequently used methods of preparation for treatment of infertility in males as presented in Fig. 3.

**Fig. 4:**
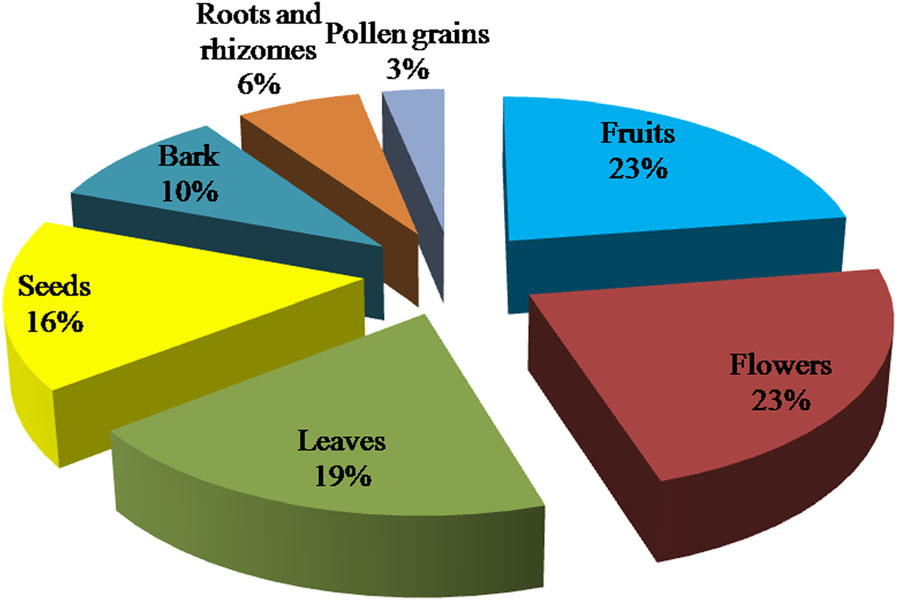
Frequency of parts used from the utilized plants in the treatments of infertility in females in the West Bank

Flowers, fruits, leaves, and seeds were reported to be the most frequently used parts of plants for the treatment of infertility in females, constituting about 65% of the parts used. This was followed by bark, roots, rhizomes and pollen grains as shown in Fig. 4.

**Fig. 5:**
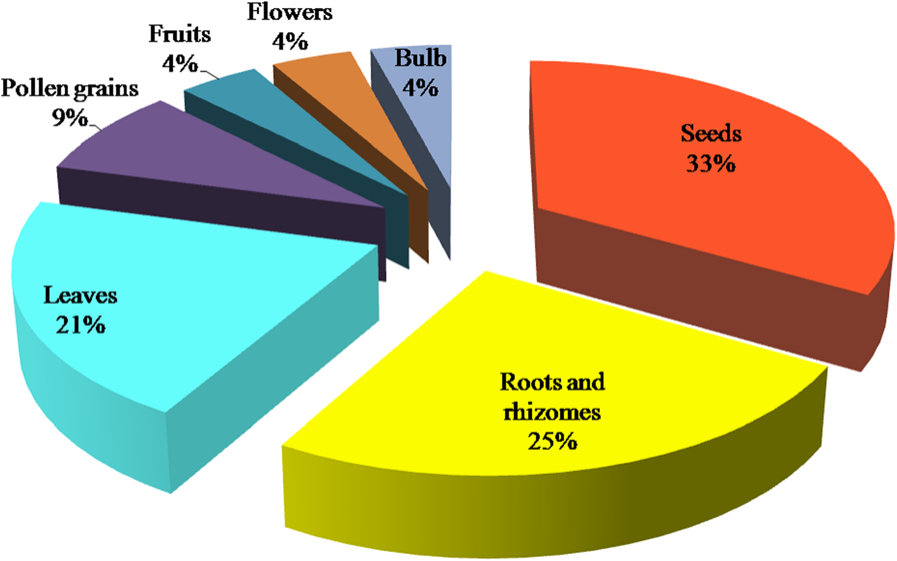
Frequency of parts used from the utilized plants in the treatments of infertility in males in the West Bank

Correspondingly, seeds, roots, leaves, and pollen grains were reported to be the most frequent parts used of plants in the treatment of infertility in males, constituting about 88% of the parts used. This was followed by fruits, flowers, and bulbs as can be seen in Fig. 5.

The utilization of herbals in the treatment of various physiological disorders and diseases goes back to several millennia and more than 60% of the world’s population, especially in the rural areas of developing countries, utilized plants, and other natural products for pharmaceutical and medical aims. In addition, about 25% of modern medications are derived from herbal products and nowadays, the use of natural herbal products has shown an increase in both developed and developing countries [26, 27]. For huge numbers of childless people, infertility disease considered a personal social and psychological problem, which equitably distributed between males and females. Hence, since ancient times, herbal remedies are considered one of the most available methods in the treatment of this disorder [28]. Recently, various isolated natural compounds or crude plants extracts are widely used in the treatment of infertility in males such as low sperm accounts, sexual asthenia, erectile dysfunction, the absence of libido and other psychological and physiological disorders [29].

Concerning the fertility in females, it may be affected by many factors, including various physiological diseases and disorders, malnutrition, and malformations of the uterus [14].

Usually, the selection of herbs and remedies by traditional healers is based on their experience and information that have been inherited from their ancestors. As shown in Table 1, the majority of the informants (traditional healers) were females and most of them were uneducated people. This result may be due to the Palestinian culture where parents try to transfer the acquired knowledge to their children. In addition, the reason why the majority of them were uneducated may be due to the bad economic situation in this country so many of them consider this tradition as a source of economic income. Accordingly, the results may be of scientific value and good credibility in some way, since these informants may be struggling to find the best results in order to maintain their clients. The same table also showed that high percentages of the informants were from the rural area of Jericho. This region is considered a very old historical profile as well as it considered the oldest city and the lowest area in the entire world.

The results of the current study showed that the Palestinian ethnopharmacology is rich in herbal remedies used in the treatment of infertility in males and in females.

In fact, a huge number of plants families (44 families) were reported to be effective in the treatment of this disorder. However, different plants species were used to treat infertility in females and males as reported in Tables 2 and 3. In addition, Table 4 showed the highest FC and CV values for the herbal remedies used in the treatment of infertility among females and males in the West Bank area of Palestine.

**Table 4:** The most cited herbal remedies used in the treatments of infertility in females and males

Infertility	Herbal remedies		
Males	*Ferula hermonis* roots infusion	*Phlomis brachyodon* leaves decoction	*Phoenix dactylifera* pollen grains paste
Female	*Ceratonia siliqua* pollen grains powder	*Anastatica hierochuntica* fruits infusion	Parietaria Judaica leaves decoction

Moreover, in the case of infertility in females, Table 2 showed that 31 herbal remedies were used in the treatment of this disorder and most of them were obtained from fruits and flowers, while the most used method of preparation was an infusion. The same table also showed that the highest Frequency of Citation (FC) of herbal remedies in case of female infertility was 98.04% for pollen grains from *Ceratonia siliqua*, 88.24% for *Anastatica hierochuntica* fruits and 84.31% for *Parietaria judaica* leaves. Moreover, Table 3 showed that 24 plants prescribed by traditional healers in the West Bank area of Palestine for the treatment of infertility in males and most of them obtained from the plant’s seeds, whereas as the most used method of preparations were decoctions. Meanwhile, the highest Frequency of Citation (FC) of herbal remedies in case of infertility in males were 96.08% for *Ferula hermonis* roots, 88.24% for *Phlomis brachyodon* leaves and 86.27% for *Phoenix dactylifera* pollen grains.

In comparison with other regional ethnopharmacological surveys about herbal remedies used for the treatment of infertility, Palestine is considered the richest region with these remedies.

In fact, 11, 13 and 23 plants used in the treatment of infertility in males and females were reported in the Lebanese, Jordanian and Egyptian folk medicine respectively [30–32].

Table 2 showed that the highest Frequency of Citations of herbal remedies which used in case of infertility in males were 96.08% for *Ferula hermonis* roots, 88.24% for *Phlomis brachyodon* leaves and 86.27% for *Phoenix dactylifera* pollen grains.

Throughout literature review, in the neighboring countries as Lebanon and Jordan, *Ferula hermonis* was found to be used in case of infertility in males [33–35].

Moreover, Hadidi *et. al.* and by Zanoli et al., investigated the potential positive effect of *F. hermonis* on male’s infertility. Both groups of the study concluded that the acute use of *F. hermonis* cause an increase in male fertility while the prolonged use of this plant may lead to fertility disturbances. Moreover, the study conducted by Zanoli et al showed a reduction in body weight and in weights of testes and other sex accessory organs [36, 37]. Another study that was conducted by Homady et al. confirmed the aphrodisiac effect of *Ferula hermonis* in male and female mice after intragastric application of 3 mg/kg/day of this plant extract for 6 weeks [38].

Meanwhile, *Phlomis brachyodon* did not mention in any folk medicine for the treatment of male’s infertility also its pharmacological effect not verified yet. Moreover, the pollen grains of *Phoenix dactylifera* has been used in the treatment of infertility in males in the folk medicine of several Arabian countries [39, 40].

Another study that was conducted by Bahmanpour et al. and Abedi et al., demonstrated that *Phoenix dactylifera* pollen grains extract can improve sperm parameters and reproductive system in adult male rats [41, 42]. However, to the best of our knowledge, no sufficient scientific studies were conducted in order to prove the safety of consumption of pollen grains of *Phoenix dactylifera* plant except that one which was conducted by Sadiq et al [43].

Moreover, the highest Frequency of Citation (FC) remedies which used in case of infertility in female were 98.04% for pollen grains from *Ceratonia siliqua*, 88.24% for *Anastatica hierochuntica* fruits and 84.31% for *Parietaria judaica* leaves.

To the best of the authors’ knowledge, it has not been reported in previous studies about folk medicine, evidence-based uses and toxicity of the pollen grains of *Ceratonia siliqua*, the fruits of *Anastatica hierochuntica* and the leaves of *Parietaria judaica* in the treatment of infertility in females and this study will be the first one.

Concerning the used preparation methods, infusions and decoctions were the most frequently used methods of preparation for treatment of infertility in females and males (Figs. 2 and 3). However, these two methods are considered harsh methods and may negatively affect the efficacy and the final organoleptic properties of the obtained extracts. Therefore, it would be interesting to assess the efficacy and the organoleptic properties of the most cited plants after being extracted by using the friendliest extraction methods such as the cold press and critical fluid extractors. Concerning the difference between herbal remedies that were advised for male and female, this may be due to the difference between genders in term of physiology and types and level of hormones as well as due to the phytochemical constituents that should affect this physiology and hormonal level.

The limitations of the current study are pharmacological, toxicological and clinical studies to confirm the most cited plant’s mechanism of actions, safety, and pharmacological efficacy. Such studies usually include a complete chemical analysis for identification of their different chemical constituents, especially those responsible for their pharmacological actions.

## Conclusion

Throughout history, males and females have tried to enhance or control their fertility with various levels of societal support. This study showed that the ethnomedicine in the West Bank area of Palestine is rich with plants which used for the treatment of infertility in males and females in comparison with the neighboring countries. Some of the plants found in this study are also used for treating infertility problems elsewhere while others are being reported for the first time. Also, it’s worthy to take in considerations that these plants had not been evaluated clinically to approve its safety and efficacy.

## Data Availability

The datasets used and/or analyzed during the current study available from the corresponding author on reasonable request.
